# Demographic and Phenotypic Effects of Human Mediated Trophic Subsidy on a Large Australian Lizard (*Varanus varius*): Meal Ticket or Last Supper?

**DOI:** 10.1371/journal.pone.0034069

**Published:** 2012-04-03

**Authors:** Tim S. Jessop, Peter Smissen, Franciscus Scheelings, Tim Dempster

**Affiliations:** 1 Department of Zoology, University of Melbourne, Melbourne, Victoria, Australia; 2 Department of Conservation and Research, Zoos Victoria, Parkville, Victoria, Australia; 3 Australian Wildlife Health Centre, Healesville Sanctuary, Healesville, Victoria, Australia; Australian Wildlife Conservancy, Australia

## Abstract

Humans are increasingly subsidizing and altering natural food webs via changes to nutrient cycling and productivity. Where human trophic subsidies are concentrated and persistent within natural environments, their consumption could have complex consequences for wild animals through altering habitat preferences, phenotypes and fitness attributes that influence population dynamics. Human trophic subsidies conceptually create both costs and benefits for animals that receive increased calorific and altered nutritional inputs. Here, we evaluated the effects of a common terrestrial human trophic subsidies, human food refuse, on population and phenotypic (comprising morphological and physiological health indices) parameters of a large predatory lizard (∼2 m length), the lace monitor (Varanus varius), in southern Australia by comparison with individuals not receiving human trophic subsidies. At human trophic subsidies sites, lizards were significantly more abundant and their sex ratio highly male biased compared to control sites in natural forest. Human trophic subsidies recipient lizards were significantly longer, heavier and in much greater body condition. Blood parasites were significantly lower in human trophic subsidies lizards. Collectively, our results imply that human trophic subsidized sites were especially attractive to adult male lace monitors and had large phenotypic effects. However, we cannot rule out that the male-biased aggregations of large monitors at human trophic subsidized sites could lead to reductions in reproductive fitness, through mate competition and offspring survival, and through greater exposure of eggs and juveniles to predation. These possibilities could have negative population consequences. Aggregations of these large predators may also have flow on effects to surrounding food web dynamics through elevated predation levels. Given that flux of energy and nutrients into food webs is central to the regulation of populations and their communities, we advocate further studies of human trophic subsidies be undertaken to evaluate the potentially large ecological implications of this significant human environmental alteration.

## Introduction

Humans exert an incredible array of ecological and evolutionary influences on wild animals and their ecosystems [1]. For example, production and loss (e.g. waste) of human food resources in urban, periurban, agricultural and natural landscapes represents an emergent and growing trophic subsidy for associated food webs [2]. Human trophic subsidies include any direct provision of food (i.e. energy) or associated nutrient alteration which modifies the nutritional landscape of animals [2]. For example, the aesthetic enjoyment of feeding birds leads to the introduction of a massive food subsidy to wild animals; 43 and 75% of households in the US and UK, respectively, feed birds [3], [4]. Given that the flux of energy and nutrients into food webs is a central process underpinning the regulation of animal populations and ecological communities [5], [6], human trophic subsidies may drive changes at the individual level which have community level consequences [7], [8], [9].

**Figure 1:**
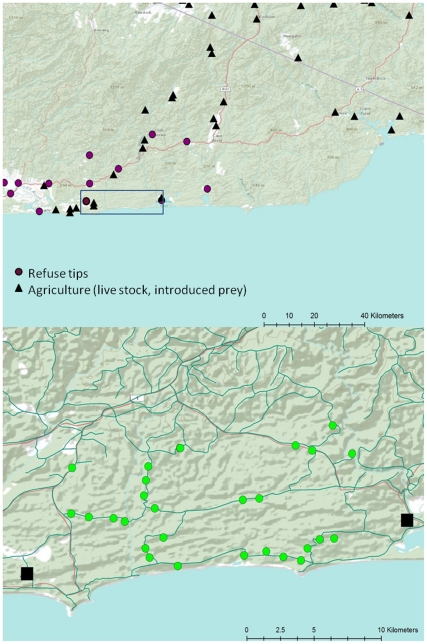
Figure (A) depicts potential sources of human mediated trophic subsides available to Lace Monitors in the general vicinity of the study site (rectangle) in southern Australia (inset). These include refuse tips and farms where domesticated animals and introduced prey (eg. rabbits) could increase food availability to lizards above, and with differenct consequences, than that sourced in natural forests. Figure (B) depicts the two refuse tips (black squares) and corresponding control sites (black polygons with green dots representing individual lizard capture locations) that were evaluated to assess the population and phenotypic consequences of human trophic subsidies on Lace Monitor lizards in southern Australia. Symbols purely represent location and not scale.

At the heart of nutrient and energy flows in food webs are the immediate functional consequences for individual consumers. Native animals that consume human trophic subsidies receive nutrition that may differ markedly in energy, nutrient composition and quality from their natural diet. Thus, via nutrition alone, human trophic subsidies could cause novel, complex and even antagonistic fitness effects for native animals [10] (Figure 1). Modified macro- (i.e. protein, fat, carbohydrates) or micro-nutrient intake might alter phenotypic traits that improve fitness, such as increasing growth rates, fat stores, reproductive effort and immunocompetence [9], [11]. Such responses could lead to higher fitness phenotypes and dramatic shifts in localized population parameters (e.g. survival, reproductive output) and in turn positively influence population growth rates. Hence, human trophic subsidies modified habitats could act as population sources [9], [12], [13].

Alternately, and analogous to the human obesity epidemic, costs may arise if human trophic subsidies provides excessive calorific intake and imbalanced nutrition causing metabolic syndromes and nutritional disorders [10], [14]. Additionally ingestion of detrimental food additives (e.g. chemicals/hormones) and exposure to novel pathogens harboured in food [15] could further increase costs to animal recipients of human trophic subsidies. Human trophic subsidies -mediated phenotypic responses are likely to be further influenced by population density dependent effects. As many sources of human trophic subsidies are spatially discreet, they serve as high density population aggregation areas, relative to low density populations inhabiting adjacent natural environs [13], [16]. human trophic subsidies induced aggregation could exacerbate social conflict and territoriality [17], skew sex ratios [18], increase parasite/disease transmission [11], elevate stress levels, decrease immunocompetence and ultimately lead to reduced individual fitness, either through decreased reproductive success or elevated mortality rates through higher predation risk. Thus, spatially discrete human trophic subsidies in natural environments could drive ecological traps, resulting in fitness costs to individuals and demographic changes in populations [19]. Robertson and Hutto [20] propose that ecological traps arise from habitat alteration that operates in by increasing the attractiveness of an environment by enhancing the set of cues that animals recognise as beneficial and concurrently decreasing the suitability of a habitat. Conceivably, animals could prefer human trophic subsidized - habitats over natural habitats as they mimic a strong ecological cue (i.e. high food availability) which typically signals the presence of a good quality natural habitat [13]. Human trophic subsidies -modified habitats have the potential to modify other ecological processes and reduce the fitness of resident animals. Under such circumstances, human trophic subsidies -modified habitats would acts as ecological traps, with consequences at the individual level ultimately leading to reduced local fitness and in the absence of sufficient recruitment from outside immigration cause negative population growth. A good example of an ecological trap is that of red necked grebes (*Podiceps grisegena*) foraging in a large scale carp (*Cyprinus carpio*) aquaculture venture [21]. Here adult grebes pending their choice of carp size within particular fish rearing ponds, where fish are raised in discreet size classes, can impact offspring survival. If adult birds choose to forage at ponds containing medium sized carp which grow to become too large during the course of egg development and incubation, it denies young birds a food supply causing high chick mortality, even though the adult condition and survival is high [21].Human trophic subsidies are common in natural landscapes, but their effects on individuals are poorly understood [16], [19]–[21]. Here, we evaluated the effects of human trophic subsidies on a common Australian predator, the Lace Monitor (*Varanus varius*). These large lizards (∼2 m length) exploit multiple sources of human trophic subsidies from agriculture (carrion from live stock or introduced prey foraging on pasture e.g. rabbits) and human food waste deposited in refuse tips or accessible in recreational wilderness areas (e.g. food from campers and hikers) (Figure 1A). Further, given that human trophic subsidies are accessible at spatially confined point-sources nested within natural landscapes, they provide good natural experiments to examine the effects of human modified nutritional input which are not confounded by other major landscape changes that could influence this predator’s ecology.

**Figure 2:**
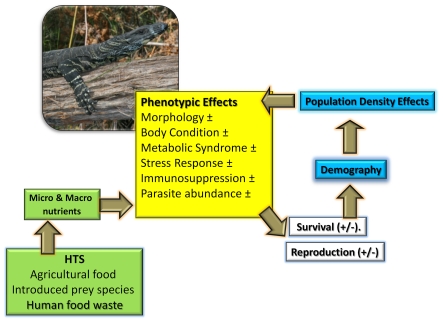
Conceptual outline of the potential phenotypic and demographic effects of human trophic subsidies on Lace Monitor lizards (photo) in a predominantly natural landscape.

Human trophic subsidies could influence these large predatory lizards via two key pathways that ultimately influence their population abundance via changes in demographic processes (Figure 2). The first pathway considers phenotypic responses of lizards to human trophic subsidies. Pending the quantity and quality of macro- and mirco-nutrients ingested, we would expect diverse changes in multiple morphological and physiological parameters. For example, spatial variation in ungulate prey resources in Komodo dragons (*Varanus komodoensis*) is associated with ∼ fourfold differences in maximum body mass [22], [23]. Hence, greater protein availability in human trophic subsidies -modified habitats may enable lizards to grow faster and larger relative to lizards in natural habitats. Similarly, for many animals, variation in body condition is often associated with large individual differences in immunocompetence, which underpins parasite resistance and regulates both basal and maximal endocrine stress levels [24]–[26].

The second pathway through which human trophic subsidies could affect Lace Monitors is via altering realized or perceived habitat quality cues. Increased availability of protein-rich food is likely to attract Lace Monitors, who routinely consume both live prey and scavenge for carrion. Thus, human trophic subsidies sites could continuously recruit lizards from adjacent habitats. However, the net consequence of human trophic subsidies recruitment could be either positive or negative for the local demography pending how individual lizards interact with conspecifics. For example, prolonged focal supplementary feeding of Komodo dragons to enhance tourism (i.e. ease of viewing of this large predator) increased local population densities of lizards by nearly sixfold relative to adjacent natural areas [27]. However, such aggregations were dominated by large male lizards (the larger sex) that skewed the operational sex ratio relative to natural areas. A strong body of research and theory indicate that increased competition for mates leads to skewed or reduced reproductive success for males [28]–[30]. A habitat-related cost of supplementary feeding via human trophic subsidies to lace monitors could also result in shifts to operational sex ratios.

To evaluate these potential effects of human trophic subsidies, via human food refuse, on Lace Monitors in Southern Australia, we used integrative methods to assess both population (counts and sex ratio) and phenotypic effects (morphology, metabolic enzymes, endocrine stress state, plasma biochemistry and parasite load).

## Materials and Methods

### Study Location

Our study area comprised four sites in East Gippsland, Victoria, Australia (37°S, 148°E). The sites were divided into two paired treatments comprising a refuse tip (hereafter referred to as human trophic subsidy) from an adjacent township (both with populations of <200 people) and a control area of natural forest (∼10 000 Ha) of adjacent forest (Figure 2b). The two human trophic subsidy sites at their existing locations had been in operation since 1992 and 1995, respectively. Like many parts of non-urban Australia, waste refuse sites are situated away from human settlements and within forest. Human food refuse was disposed into both refuse tips on a daily basis and enabled Lace Monitors to consume an ongoing food supply. Given the remoteness of these tips from major human population centres, refuse was largely household and not industrial or medical in nature. Direct observations of lizards feeding within human trophic subsidy sites indicated diets were dominated by meat from domestic livestock and fish. In contrast, diets at control sites in this system were dominated by the marsupials ringtail possum (*Pseudocheirus peregrinus*; ingested whole) and swamp wallaby (*Wallabia bicolour*; eaten as carrion; [31]). These two prey species accounted for 95% of natural diets by weight.

All human trophic subsidized and control sites (hereafter referred to as treatments) were separated by a buffer of 5 km to ensure dietary independence among lizards within their respective treatments. This degree of spatial independence among sites was designed to exceed the largest home range estimates calculated for this species [32]. Animal captures across all sites were conducted concurrently during January/February of 2009 coinciding with the peak activity of these lizards during summer.

### Population Counts

To estimate Lace Monitor density within control and human trophic subsidized areas, we conducted visual count surveys along fixed transects over six occasions in January and February 2009. All surveys were conducted at 10 km hr^−1^ from a vehicle and search distances were constrained to 30 m either side of the survey mid line to standardise search effort. In control forest areas, where goannas foraged on a natural diet, we established two survey loops (40 and 38 km; total survey effort of 430 km) from which we counted goannas. At the two human trophic subsidized sites, we conducted visual surveys along a 0.5 km transect running through the midline of the refuse property. All counts were conducted on clear warm days (26–30°C) to maximize and standardise detection of monitor lizards. Similar visual census methods have been used elsewhere to measure numerical trends in varanid populations [33].

### Lace Monitor Captures

To compare the sex ratios and phenotypic consequences for Lace Monitors feeding on natural diets and human trophic subsidies derived from human waste food, we captured lizards with a noose pole from the two human trophic subsidized sites (human trophic subsidized site 1 = 20 lizards; human trophic subsidized site 2 = 13 lizards) and two adjacent control sites (Control 1 = 14 lizards; Control 2 = 13 lizards) (Figure 2) in January and February 2009. Following capture, Lace Monitors were restrained, measured and weighed. Each lizard captured was inserted with a passive integrated transponder in the right hind leg. In addition we painted each lizard’s back with a unique paint code (using non-toxic fabric marker) to increase our ability to recognise individual lizards and make general observations of their behaviour whilst conducting our study. All animals were subsequently sampled for sex and phenotypic consequences as follows.

### Sex Ratio

Genomic DNA was extracted from blood in lysis buffer using a DNeasy Blood and Tissue Kit (Qiagen), following the manufacturer’s protocol. To determine potential sex ratio differences between treatments, lizards from which blood samples were taken were genetically sexed using PCR primers that amplified sex specific alleles [34]. Amplifications were performed in a 20 µL total volume, containing 2 µL of DNA (diluted 1∶10 in TE buffer), 10 µL Gotaq (Promega), 0.5 µL of each primer (10 µM) and 7 µL of H_2_O. PCR amplifications were performed on a Corbett Palm-Cycler using a touchdown thermal cycle program with the following parameters: initial denaturation @ 94°C for 5 mins, followed by two cycles of 94°C for 30 sec, an annealing step @ 65°C for 30 sec and 72°C for 90 sec; 2 cycles each with annealing temperatures of 60°C, 55°C and 50°C; 30 cycles with an annealing temperature of 48°C; then a final extension step of 10 mins @ 72°C. Amplifications were checked on a 1.2% agarose gel and amplification patterns were compared to those of a male and female whose sex had been verified anatomically.

### Phenotypic Consequences of Human Trophic Subsidies on Lace Monitors

To test if the phenotypic consequences for Lace Monitors feeding on human trophic subsidies derived from human waste food differed from those feeding on natural diets, blood was sampled (3 ml of blood) from the caudal vein using a syringe within 3 minute of capture to enable baseline parameters. Half of the blood was immediately transferred to a lithium heparin container (BD Microtainer™ Tubes, BD Vacutainer Systems, Franklin Lakes, NJ) and the other half into a plain container (BD Microtainer™ Tubes, Vacutainer Systems, Franklin Lakes, NJ). In the field, blood was stored at 4°C in a portable insulated ice box and once back at camp samples were prepared for analysis. To prevent re-capture and sampling, lace monitors were individually identified with a paint-code on their back and implanted with PIT tags (TROVAN® ID-100BC, Microchips Australia Pty Ltd, Australia) subcutaneously in the dorsal right thigh. We measured multiple parameters to evaluate the effects of human trophic subsidies on lizard phenotype and population abundance. These included:

### Morphology

Body size (snout to vent length) and mass of each individual was recorded and body condition was calculated using the residuals from the regression equation of mass (natural log transformed) plotted against body length (using snout to vent length, natural log transformed). Transformed data reduced the influence of changes in body shape during ontogeny and thus eliminated allometric differences in body condition.

### Haematology

We measured the red blood cell packed cell volume (PCV) using standardized measurement protocols post standard centrifugation of microhematocrit tubes. A leukocyte differential count was performed via examination of air-dried, whole blood films on a microscope slide stained with Romanowsky stain (Rapid Diff, Australian Biostain Pty Ltd, Traralgon, Vic). Although some authors like to distinguish azurophils from monocytes, their cytochemical and ultrastructural characteristics are often similar and as such they should be considered as the same cell type [35]–[37]. Therefore leukocytes were classified as heterophils, lymphocytes, eosinophils, basophils or monocytes. Heterophil/eosinophil counts were performed manually using a hemocytometer and a Unopette (Unopette®, BD Vacutainer Systems, Franklin Lakes, NJ) designed for counting eosinophils. The total white blood cell (WBC) count was then calculated by correcting the manual count for the percentage of heterophils and eosinophils present [35]. All hematological evaluation was performed by the third author in order to maintain consistency.

### Metabolic Syndrome Enzymes

Excess caloric intake often induces multiple pathologies including metabolic syndromes. Elevation of aspartate aminotransferase (AST) and creatinine kinase (CK) is indicative of hepatic and cardiac associated metabolic syndrome in vertebrates. Both enzymes were quantified using 100 μl of serum loaded onto an avian-reptilian biochemistry rotor on the Vet Scan analyser (Abaxis, Inc. Union City, California 94587, USA).

### Plasma Biochemistry

Diet has a major influence on the biochemistry of reptiles and blood biochemistry is often used to assess their physiological status [35], [36]. For example, hyperuricemia may occur in animals that have recently ingested a meal high in protein. In general, carnivorous reptiles have higher blood uric acid concentrations than herbivorous reptiles [35]. Similarly, hyperglycemia and hyperkalemia may be indicators of excessive dietary intake of glucose and potassium respectively). In contrast, hypocalcemia, hypoproteinemia and hypoalbuminemia are commonly associated with chronic malnutrition [35]. To determine the effect on an human trophic subsidized diet on Lace Monitors, we compared serum taken from animals in the two treatments for albumin, calcium, glucose, potassium, sodium, phosphorous, total protein and uric acid. Serum biochemical testing was performed using the avian-reptilian rotor on the Vet Scan analyser. All samples were run using 100 μl of serum.

### Plasma Stress Hormone

We measured total plasma concentrations of corticosterone using a commercially available RIA kit (MP Biomedical, Ohio, United States of America) and associated protocol [38]. Samples were analysed across two assays while a small number of capture stress samples served as a positive control for corticosterone. Preliminary assays determined that the sufficient volume of sample plasma to be used for assay was 25 µL for corticosterone. To reduce cross reaction and interference all samples were individually extracted using two washes of diethyl ether. To measure the efficiency of extraction 20 μL (≈2000 cpm) of tritiated corticosterone was added to each sample prior to extraction. To estimate steroid extraction efficiency, 50 μL of each extracted sample was placed into a scintillation vial containing 2 mL of scintillation fluid (Ultima Gold). Sample radioactivity was estimated using a Beckman 2100R Liquid Scintillation Counter. The extraction efficiency for each sample was calculated as the quotient of radioactivity (CPM) remaining in the sample relative to the total amount of radioactivity added to each sample pre-extraction (determined from extraction controls).

Final steroid concentrations were calculated from standard curves and corrected for individual sample recovery, individual plasma volume and the addition of tritiated steroid. Average (±SE) sample recovery was 75.7%±0.028 with an intra-assay coefficient of variation of 7.6% and an inter-assay coefficient of variation of 13.04%.

### Parasites

The prevalence of a haematozoan parasite (*Haemogregarina varanicola*) infecting erythrocytes were examined manually from blood smears. The frequency of parasites within 500 erythrocytes (1000×magnification) was used as our measure of parasite load. Haematozoan parasites are known to negatively influence fitness attributes in other large reptiles [39]. All hematological evaluation was performed by the third author in order to maintain consistency.

### Statistical Analysis

We analysed data using generalized estimating equations (GEE) with Gaussian (phenotypic measures), binomial (sex-ratio), or Poisson distribution (population, white blood cells & haemoparasite counts) and their respective link functions, conducted in SPSS 19. For GEEs, we included either site or transect number as a random effect in our analyses. As few females were captured and we had no *a priori* information to suggest human trophic subsidies would differentially affect male or female morphology or physiology, we pooled the sexes for analyses.

## Results

### General Observations of Foraging Behaviour

Within natural control sites we didn’t reencounter the same individual lizards after inital capture. However at human trophic subsidized sites we routinely obseserved the same individuals (recognised by paint codes on their backs) across the 4 weeks of study both residing and foraging on food refuse at human trophic subsidized sites. Repeated observations of the same individuals suggested that the composition of lizards captured at human trophic subsidized sites were relatively stable. Pending the nature and amount of garbage deposited daily we observed between 2–9 Lace monitors concurrently engaged in foraging. Whilst we could not explicitly estimate the daily amount of food ingested by individual lizards it was evident by the frequency of feeding that nutritional requirements were either met entirely or largely by food refuse. As when not foraging on refuse, lizards were readily observed to bask both on logs, and in closely adjacent trees suggesting that they were largely sedentary forsaking normal foraging activities to maintain strong spatial fidelity to human trophic subsidized sites.

### Population Counts

**Figure 3:**
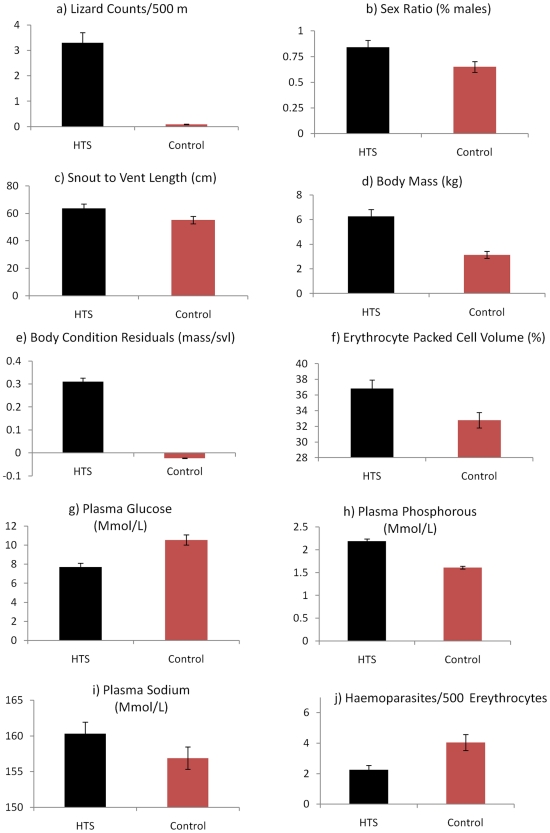
Statistically significant treatment effects of human trophic subsidies, arising from human food refuse, on population and phenotypic measures in Lace Monitor lizards.

Population surveys revealed that human trophic subsidized modified habitats had significantly higher counts of lace monitors compared to control sites (; Wald χ^2^_1,22_ = 26.0; *P*<0.001; Figure 3a).

### Sex Ratio

The sex ratio of lizards was 2.8 times more male-biased at human trophic subsidized sites (5.3∶1 male:female) than control sites (1.9∶1; Wald χ2_1,58_ = 6.5; P = 0.011; Figure 3b).

### Morphology

Lace Monitors captured from human trophic subsidized areas were significantly longer (Wald χ^2^_1,58_ = 25; *P*<0.001; Figure 3c), over twice as heavy (Wald χ^2^_1,58_ = 36; *P*<0.001; Figure 3d) and in better body condition (Wald χ^2^_1,58_ = 10; *P* = 0.002; Figure 3e) than lizards captured in adjacent control areas.

### Haematology

Packed cell volumes of erythrocytes of Lace Monitors captured from human trophic subsidized sites were significantly greater (Wald χ^2^_1,58_ = 4.1; *P* = 0.004; Figure 3f) than lizards captured in control sites. White blood cell counts did not differ significantly between treatments (Wald χ^2^_1,58_ = 0.9; *P* = 0.34; Figure 3g).

### Metabolic Syndrome Enzymes

Plasma enzymes levels for aspartate aminotransferase (Wald χ^2^_1, 58_ = 0.3; *P* = 0.6) and creatinine kinase (Wald χ^2^_1, 58_ = 0.5; *P* = 0.5) did not differ significantly between lizards captured in human trophic subsidized and Control habitats.

### Plasma Biochemistry

There were significant treatment differences for three of the eight serum biochemistry parameters measured between human trophic subsidized and control Lace Monitors. These included: significantly lower plasma glucose (Wald χ^2^_1, 58_ = 41; *P* = 0.001; Figure 3g), higher plasma phosphorous (Wald χ^2^_1, 58_ = 113; *P* = 0.001; Figure 3h) and higher plasma sodium (Wald χ^2^_1, 58_ = 14; *P* = 0.001; Figure 3i) at human trophic subsidized sites. No significant treatment differences were observed for albumin, calcium, potassium, total protein and uric acid plasma concentrations.

### Plasma Stress Hormone Levels

There was no significant difference in basal plasma corticosterone levels (Wald χ^2^_1, 58_ = 1.6; *P* = 0.2) of lizards between treatments.

### Parasites

Lizards from the human trophic subsidized sites had significantly less haemoparasites in their blood compared to control site lizards (Wald χ^2^_1,58_ = 9.7; *P* = 0.002; Figure 3j).

### Discussion

Humans are increasingly subsidizing the nutritional dynamics of natural food webs. These subsidies have multiple direct and indirect effects on the ecology and evolution of consumers [6], [7]. Here, we provide evidence that provision of human trophic subsidies to large predatory lizards, via human food refuse, results in phenotypic changes to individuals which are consistent with increased nutritional intake.

Human trophic subsidized habitats were highly attractive to lizards, with the mean population count estimate thirty five times higher than control sites. This result is consistent with other studies documenting the potent effect of human trophic subsidies in driving hyper-abundance in native animal populations in small spatial areas [7], [16],[27], [40], [41]. Further, all morphological and phenotypic parameters investigated suggested individuals benefitted from the human trophic subsidies; lizards at human trophic subsidized sites were significantly larger and heavier than controls, lacked elevation in key enzymes used to infer metabolic syndromes, showed no significant elevation of plasma corticosterone (a marker of stress), and had reduced blood parasites loads of *Haemogregarina varanicola*. Differences in serum biochemistry were evident, and potentially suggested that differences in dietary intake and endogenous state could be at play. For example, human trophic subsidized lizards had significantly greater plasma phosphorous and sodium concentrations, a feature consistent with consumption of human derived and highly processed foods. In contrast, lizards in control sites had significantly higher plasma glucose levels, significantly decreased body condition and trended towards higher basal corticosterone levels. Collectively, these measures often signal reduced food intake, or increased activity [26]. Either explanation is plausible given that control lizards are likely to be more free-ranging, active and less well fed than lizards occupying well resourced human trophic subsidized sites. These results suggest that the nutritional advantages provided by human trophic subsidies had potent effects on either attracting individuals or increasing localized rates of fecundity and survival [13], [16].

What are the population-level implications of localized hyper-abundance comprising individuals biased towards high quality phenotypes? Increased body size, better body condition and reduced parasite load should promote survival and influence reproductive success via offspring quantity and quality. Similarly, it is conceivable that such focal food resources attract high fitness genotypes that are better able to compete within high density aggregations. Together human trophic subsidies could invoke rapid, localized selection for high fitness individuals which contribute greater reproductive output and hence population dynamics in human trophic subsidized areas may be dissimilar to adjacent natural populations [13].

Hyper-abundance, driven by human trophic subsidies, could influence the demography and ensuing functional role of this predator in adjacent forest, with negative population consequences for specific prey species. Ringtail possums are the main prey item of lace monitors in this system [31] and their numbers may be driven down if goannas switch between human trophic subsidies and natural prey. Analogous circumstances exist for feral cat hyper-abundance around human trophic subsidies, with small prey species suppressed [42].

Offsetting the phenotypic benefits gained by individuals aggregating at human trophic subsidized sites, however, is the highly male-biased sex ratio that results. This suggests that food subsidized habitats may preferentially attract males (the larger sex) who presumably monopolise food at the expense of smaller females. A consequence of a highly male-biased sex ratio is increased competition for mates, which would potentially reduce average male reproductive success [30]. Further, despite driving hyper-abundance and elevating phenotypic condition for adults, human trophic subsidized sites may conceivably adversely affect offspring survival if young are subject to higher predation levels. Municipal waste sites attract a suite of other predators, such as red foxes [43] and raptors, which are known to prey upon either eggs or juvenile goannas [44]. In addition, goannas are cannibalistic, and thus high density aggregations at human trophic subsidized sites might result in high juvenile mortality. If the human trophic subsidized habitats attract and draw in lizards from the surrounding natural forest, and their offspring are subject to elevated rates of predation, an ecological trap could ensue if the population is driven down.

Our results could infer a fitness trade-off whereby phenotypic quality promotes survival, but reproduction is generally decreased because of highly male-biased populations. However, without ultimate measurements of fitness, the consequences of human trophic subsidized sites nested within natural environments to lizard populations remain unknown. Hence, it is necessary to conduct longer term studies to fully understand the consequences of human trophic subsidies for lizard populations across the landscape.

Community ecologists have long understood the importance of natural fluctuations in energy, material and organisms that constitute allochthonous inputs or spatial subsidies for regulating ecosystem function [5], [6]. However, such studies have focused on population level effects principally around abundance and range shifts of species recipient to such subsidies. The advent and ongoing rise of human trophic subsidies that can constitute multiple, but often novel sources of nutrition from highly modified waste food, domestic crops and animals, poses emergent and potentially strong selection on animals. Subsequently, human trophic subsidies could have profound effects on individual fitness and ultimately the demography of populations. These effects could then cascade through community dynamics and synergistically interact with other human landscape altering processes [9]. Our study has touched on a single, yet common human trophic subsidy (within a landscape with several) for a single species and illustrated multiple phenotypic and demographic consequences. As landscapes become increasingly modified by interactions between global change and human activities, we advocate that both pure and applied ecologists utilise integrative research approaches to evaluate the influences of human trophic subsidies on ecological dynamics at different scales of organization.
